# Human neuroglobin protein in cerebrospinal fluid

**DOI:** 10.1186/1477-5956-3-2

**Published:** 2005-02-25

**Authors:** Begona Casado, Lewis K Pannell, Gail Whalen, Daniel J Clauw, James N Baraniuk

**Affiliations:** 1 Division of Rheumatology, Immunology & Allergy, Georgetown University, Washington DC 20007-2197, USA; 2 Laboratory of Bioorganic Chemistry, National Institute of Diabetes and Digestive and Kidney Diseases, National Institutes of Health, Bethesda, Maryland 20892-0508, USA; 3 Department of Medicine, Division of Rheumatology, University of Michigan, Ann Arbor 48109, MI, USA; 4 Dipartimento di Biochimica A. Castellani, Universita di Pavia, Pavia 27100, Italy

## Abstract

**Background:**

Neuroglobin is a hexacoordinated member of the globin family of proteins. It is predominantly localized to various brain regions and retina where it may play a role in protection against ischemia and nitric oxide-induced neural injury. Cerebrospinal fluid was collected from 12 chronic regional or systemic pain and 5 control subjects. Proteins were precipitated by addition of 50% 0.2 N acetic acid, 50% ethanol, 0.02% sodium bisulfite. The pellet was extensively digested with trypsin. Peptides were separated by capillary liquid chromatography using a gradient from 95% water to 95% acetonitrile in 0.2% formic acid, and eluted through a nanoelectrospray ionization interface into a quadrapole – time-of-flight dual mass spectrometer (QToF2, Waters, Milford, MA). Peptides were sequenced (PepSeq, MassLynx v3.5) and proteins identified using MASCOT ^®^.

**Results:**

Six different neuroglobin peptides were identified in various combinations in 3 of 9 female pain subjects, but none in male pain, or female or male control subjects.

**Conclusion:**

This is the first description of neuroglobin in cerebrospinal fluid. The mechanism(s) leading to its release in chronic pain states remain to be defined.

## Background

The protein constituents (proteome) of cerebrospinal fluid (CSF) are altered in disease states such as meningitis, but may also be more subtly altered in many other neural conditions. CSF has been difficult to investigate because of the need for invasive lumbar punctures and the small volumes of CSF available for analysis. This situation is now rapidly changing as methods requiring microliter volumes and sophisticated analysis tools such as proteomics become available [1,2]. Proteomics has made it possible to identify scores of proteins that have not been previously discovered in this fluid.

One such protein is neuroglobin. Neuroglobin is a recently identified member of the globin family. It binds oxygen with an affinity between that of myoglobin and hemoglobin [3,4]. Neuroglobin is 151 amino-acids long with a molecular mass of ≈ 17 kDa. The mouse and human genes are 94% identical. Neuroglobin is an ancient protein (estimated < 550 Myr old) that is more related to the annelid *Aphrodite aculeate *intracellular globin (30% identify) [5] than to vertebrate myoglobin (<21% identity) and hemoglobin (<25% identity) [3]. Human neuroglobin mRNA is predominantly expressed in brain with high signal in the frontal lobe, subthalamic nucleus and thalamus. The concentration is estimated to be less of 0.01% of the total brain protein content [3]. Neuroglobin protein has not been previously detected in cerebrospinal fluids.

## Results

### Neuroglobin Peptides

Five peptides derived from neuroglobin (NCBInr ID accession 10864065) were identified using both the MASCOT software with NCBInr database and ProteinLynx Global Server with SwissProt database (Table 1). Two precursor ions with mass/charge (M/Z) ratios of 423.97 and 435.82 were identified in CSF sample #3 with the SwissProt, but not NCBInr, searches. Table 1 shows the mass-over-charge (m/z), charge state, elution time, position, sequence, molecular weight (M_r_) for each peptide. The numbers of matching peptides were 5 in sample #1, 2 in sample #2, and 3 in sample #3. The peptides mapped 31.1%, 13.9% and 17.2%, respectively, of the total neuroglobin protein sequence. They matched amino acids 1–10 and 15–30 of the N-terminal and 131–151 of the C-terminal. Trypsin digestion missed cleavage sites at amino acids 18 and 146. Reproducibility was demonstrated by the consistent retention times for the same peptides from different subjects. No low abundance neuroglobin peptides were found in other samples. BLAST sequence analysis of all six peptides identified only one protein: hypothetical 16.9 kDa protein (neuroglobin: NREF and iProClass NF00135839; SwissProt/TrEMBL Q9NPG2).

**Table 1:** Amino acid sequence of neuroglobin peptides identified from human CSF using CapLC nanoESI Q-TOF tandem mass spectrometry.

**#^a^**	**Amino Acids**	**M/z ^b^**	**Z^c^**	**Molecular Weight**		**Δ^d^**	**Amino Acid Sequences**	**Time^e ^(min)**
							
				**Calc.**	**Exp.**			
1	1–10	423.98	3	1268.65	1268.91	0.25	(-)MERPEPELIR(Q)	37.02
1	15–30	580.73	3	1738.95	1739.18	0.23	(R)AVSRSPLEHGTVLFAR(L)	42.94
1	19–30	442.99	3	1325.71	1325.95	0.25	(R)SPLEHGTVLFAR(L)	42.68
1	131–146	580.04	3	1736.87	1737.10	0.24	(R)AAWSQLYGAVVQAMSR(G)	68.23
1	131–151	761.42	3	2281.06	2281.23	0.17	(R)AAWSQLYGAVVQAMSRGWDGE(-)	74.49
2	131–146	580.02	3	1736.87	1737.03	0.16	(R)AAWSQLYGAVVQAMSR(G)	68.24
2	131–151	761.37	3	2281.06	2281.09	0.03	(R)AAWSQLYGAVVQAMSRGWDGE(-)	74.54
3	1–10	423.97 ^f^	3	1268.65	1268.88	0.23	(-)MERPEPELIR(Q)	37.31
3	15–30	435.82 ^f^	4	1738.95	1739.27	0.32	(R)AVSRSPLEHGTVLFAR(L)	43.27
3	19–30	442.99	3	1325.71	1325.96	0.25	(R)SPLEHGTVLFAR(L)	43.01

a. Subject Number

b. Mass / charge ratio

c. Charge

d. Difference (error) between the experimental (Exp.) and calculated (Calc.) molecular weights

e. Elution time expressed in minutes.

f. Peptides in Subject 3 identified only with ProteinLynx Global Server using the SwissProt database.

### Mass Spectrometry

**Figure 1:**
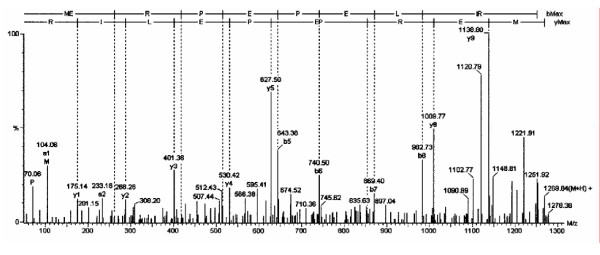
The tandem mass spectrum is shown for the neuroglobin amino acid 1 to 10 peptide. In this and the following figures, the top line represents the b-series, and the 2^nd ^line the y-series. The x-axis presents M/z and the y-axis signal intensity. The numbers are the M/z values for each daughter ion (vertical lines).

**Figure 2:**
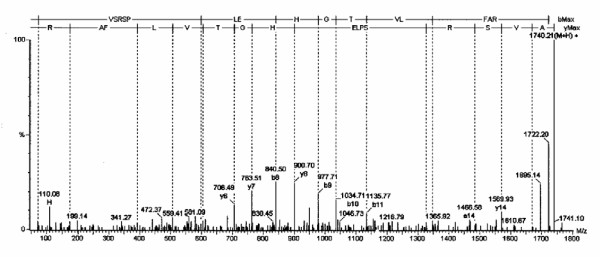
The tandem mass spectrum is shown for the neuroglobin amino acid 15 to 30 peptide.

**Figure 3:**
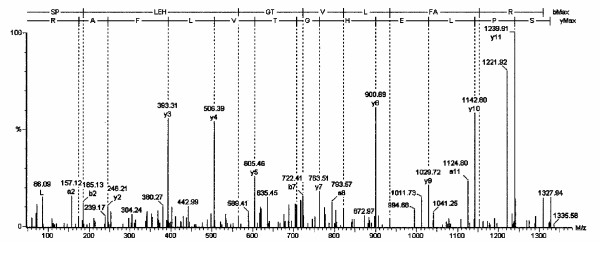
The tandem mass spectrum is shown for the neuroglobin amino acid 19 to 30 peptide.

**Figure 4:**
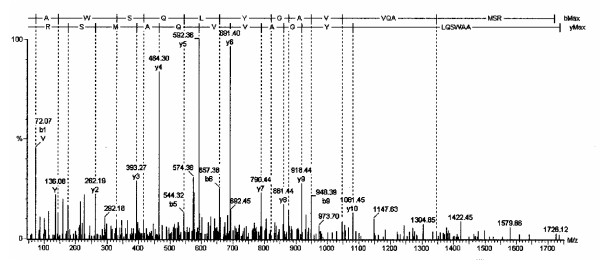
The tandem mass spectrum is shown for the neuroglobin amino acid 131 to 146 peptide.

**Figure 5:**
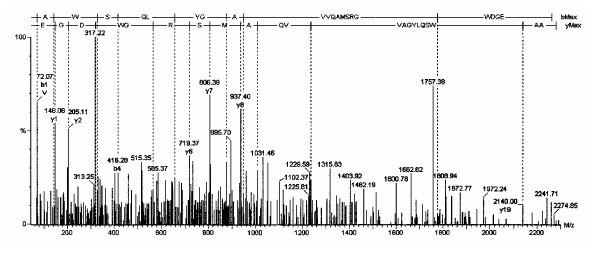
The tandem mass spectrum is shown for the neuroglobin amino acid 131 to 151 peptide.

Figures 1 through 5 show the tandem MS data for the 5 [M+H^+^] precursor ions. The mass of each b- and y-fragment is listed. The amino acid sequence is shown at the top of each spectrum using the Roepstorff nomenclature [11]. The amino acid sequences were determined from both the N- and C-terminal directions. Figures 1 and 4 show two spectra from subject #1. Figures 3 and 5 show two spectra from subject #2. Figure 2 shows one spectrum from subject #3. Neuroglobin peptides were detected in 2 of the 3 CapLC runs for subjects #1 and #2, but only in 1 run for subject #3.

The peptides with M/z of 580 and 761 (Table 1) overlapped with the 761 ion having a missed tryptic cleavage point at Arg^146^. Additional peptides were not identified, perhaps because we did not reduce disulfide bridges to reveal additional trypsin digestion sites. However, all the identified peptides were specific for neuroglobin, and so were appropriate markers for fast identification of this protein.

### Neuroglobin and Pain Subjects

Neuroglobin-derived peptides were found in 3 of 9 female pain subjects, but none of the 3 male pain subjects; it was not detected in the 3 female or 2 male control subjects. Within the chronic pain group there was no association between the presence of neuroglobin and clinical factors such as age, extent or duration of pain, or tenderness to pressure. No hemoglobin or cytoglobin [12,13] were detected.

## Discussion

This is the first description of neuroglobin protein in the CSF of any species. Neuroglobin joins cytoglobin (histoglobin) in a new globin subfamily that forms hexacoordinated heme iron complexes [12,13]. These are distinct from the pentacoordinated hemoglobin and myoglobin.

The source of neuroglobin in the CSF is likely to be brain regions such as the subthalamic nuclei (60% of total brain neuroglobin mRNA expression), frontal lobe, thalamus, occipital pole, pituitary gland, and medulla oblongata [3,14]. Immunohistochemistry confirmed this distribution with strong staining in the hippocampus, thalamus, hypothalamus (especially the paraventricular nucleus) and brainstem nuclei of cranial nerves [15]. Expression was often patchy within these regions indicating that only select neurons expressed neuroglobin. Regions with high sensitivity to hypoxia such as the cerebral cortex had constitutive expression [15]. Spinal cord was a less likely source since its neuroglobin mRNA expression was less than 10% of that from the subthalamic nuclei. Neuroglobin mRNA was expressed in the retina [16] and in peripheral nerves suggesting that the mRNA was axonally transported and translated to distal neurons [17]. The protein has a cytoplasmic distribution [18]. Neuroglobin could provide oxygen for high energy consuming processes such as synaptic activity, neural plasticity, or efferent transmitter release as in nociceptive nerve axon responses.

Neuroglobin mRNA was also present in adrenal cells and the β cells of the pancreatic islets of Langerhans [14]. Roles in diabetes or hypoxia-induced insulin secretion are unstudied. These studies of mRNA expression should not be extrapolated into relative levels of protein expression or neuroglobin turnover since concordance between microarray and proteomic studies can be as low at 13% [19].

Neuroglobin is likely to serve as an intracellular oxygen depot to facilitate oxygen diffusion to the mitochondria. A role in oxygen supply was supported by the very high expression of neuroglobin mRNA in retinal neurons but not the supporting ocular epithelium and other structures [16]. Retinal neuroglobin concentrations were estimated at > 100 μM, compared to > 1 μM for the whole brain. The retinal and muscle oxygen tensions, oxygen affinities and tissue concentrations of neuroglobin and myoglobin were comparable suggesting that the two play homologous roles in their respective tissues.

Neuroglobin might act in certain circumstances to limit neural cellular damage during hypoxia. Neuroglobin expression was inversely correlated to the sensitivity of the brain regions to ischemia [3]. For example, neuroglobin expression was 4 times higher in the cerebral cortex than the hippocampus, corresponding to the time for ischemia to cause half-maximal damage (19.1 and 12.7 min, respectively) in these tissues [20]. Neuroglobin-immunoreactive material was upregulated in the cytoplasm of neurons that were destined to survive acute cerebral ischemia, and was reduced in apoptotic neurons [21]. Hypoxic induction of neuroglobin was blocked by the mitogen-activated protein kinase/extracellular signal-regulated kinase kinase inhibitor PD98059 [22]. Like hemoglobin and myoglobin, hemin increased neuroglobin 4-fold through a separate signalling process mediated by protein kinase G and soluble guanylate cyclase. Hypoxia-inducible neuroprotective factor (HIF-1) that can induce β-globin production may play a role in neuroglobin induction. It is not clear if there are differential responses to intermittent, recurrent, or chronic cerebral ischemia.

Neuroglobin was also colocalized with nitric oxide synthase in the lateral tegmental nuclei, stria terminalis, habencule, nucleus of the tractus solitarius, periaqueductal grey matter, amygdala and subfornic organ [23]. The protein may act as a nitric oxide scavenger, a role that has also recently been proposed for myoglobin [24]. This function would protect against nitric oxide – induced damage that is part of hypoxia – ischemia related neuron injury. Nitric oxide appears to bind to the hexacoordinated deoxy ferrous form (F8His-Fe^2+^-E7His) and displace the protein from the globin [25]. This affinity may be a double-edged sword, since neuroglobin, hemoglobin and myoglobin may protect *Plasmodium *and *Trypanosoma *from the antiparasitic effects of nitric oxide [26]. Neuroglobin may also play a protective role in carbon monoxide poisoning [27].

In this study, neuroglobin was qualitatively identified in CSF from 3 female subjects with chronic pain conditions. Females have greater pain sensitivity to pressure and other stimuli (lower pain thresholds) [28], but pain is not thought to induce neural hypoxia or any of the known triggers of neuroglobin expression [21].

It is tempting to speculate that the source of neuroglobin in our samples was from nuclei involved in pain transmission or regulation such as the thalamus, prefrontal cortex, amygdala, or spinal cord dorsal horn somatic pain synaptic regions (e.g. layers 1 and 2 of Rexed). The fact that neuroglobin was not detected in any of the control females in our study makes it unlikely that the expression was related to gender. Examination of additional normal and chronic pain subjects is underway to determine the factors that may be responsible for neuroglobin expression. It is also possible that the proteomic detection of neuroglobin varies depending upon sample preparation, signal-to-noise ratio for relatively low abundance proteins compared to albumin and immunoglobulins that are present in high abundance, duration of storage, factors related to trypsin digestion, capillary liquid chromatography, mass spectrometry or bioinformatic neuroglobin peptide detection. These technical factors are unlikely to be significant since our samples were treated identically and were stored for approximately equal amounts of time.

In contrast to neuroglobin's localization, cytoglobin-immunoreactive material was localized to the cellular nucleus in all tissues examined [14]. Mammalian cytoglobin genes display an unique exon-intron pattern with an additional exon resulting in a C-terminal extension of the protein that is not present in lower species such as zebra fish [29,30]. Again, it is not clear if cytoglobin acts as an oxygen depot or sink, free radical scavenger, oxygen-sensor or transcription factor. No evidence for cytoglobin was found in cerebrospinal fluid suggesting that nuclear degeneration was not present in any of our subjects.

## Conclusion

This is the first description of neuroglobin in cerebrospinal fluid and in humans. Neuroglobin was identified in 3 of 9 female pain subjects. The role(s) for this ancient oxygen and nitric oxide binding protein in humans, and potential links to pain, remain to be fully determined.

## Methods

After obtaining informed consent, lumbar punctures were performed on 17 subjects as part of an evaluation of pain mechanisms. Twelve patients had musculoskeletal pain and five were healthy control subjects. Cerebrospinal fluid samples were aliquoted and frozen at -70°C. Lipids and peptides were extracted from 200 μl of thawed CSF by adding an equal volume of 50% ethanol, 50% 0.2 N acetic acid 0.02% sodium bisulfite ("acid-ethanol") [6]. Centrifuged pellets were reconstituted in 50 μl of 0.1 M ammonium bicarbonate buffer (pH 7.8) and digested with trypsin (protein-enzyme ratios of 20:1) at 37°C overnight. Digested peptides were separated by capillary liquid chromatography (CapLC, Waters, Milford, MA) over a Zorbax 18WSB reverse phase column (100 mm × 0.15 mm inner diameter) (Micro-Tech Scientific, Sunnyvale, CA) at room temperature for 100 min using a gradient starting at 95% solvent A (aqueous solution of 0.2% formic acid) and ending with 95% solvent B (acetonitrile with 0.2% formic acid). The elution was performed at a flow-rate of 1 μl/min.

The column eluate was pumped through a nanoelectrospray interface into a quadrapole – time of flight (Q-TOF-2, Waters, Milford, MA) mass spectrometer. MASSLYNX version 3.5 software was used to control the CapLC and Q-ToF-2, data acquisition, processing, and determination of peptide sequences. The protein identification was performed with the MASCOT MS/MS ion search software  and NCBInr protein database [7,8], and with ProteinLynx Global Server Web (Waters) with SwissProt database. The BLAST algorithm was used to compare protein queries to database sequences (e.g. Protein Information Resource, PIR, ) [9,10], proteins derived from GenBank coding sequences, and PDB atomic coordinates.

Samples were assessed by CapLC-Q-ToF-2 in triplicate. At least 2 separate peptides from neuroglobin had to identify in each individual sample to ensure that this protein, and not a related protein, was present. In an attempt to detect low abundance expression of neuroglobin peptide ions that were not selected by MS-MS (false negative results), all MS data from the appropriate CapLC retention times were reassessed at high resolution. Positive results (MS data) were checked to see if ions in MS were present but not in MS-MS for other pieces. All putative neuroglobin peptide spectra were sequenced using PepSeq (Waters) and confirmed by visual inspection.

## Competing interests

The author(s) declare that they have no competing interests.

## Authors' contributions

Casado B ^1,4^, Sample preparation, chromatography, mass spectrometry and manuscript preparation

Pannell LK ^2^, Supervision and assistance with chromatography and mass spectrometry and manuscript preparation

Whalen G ^1^, Sample preparation

Clauw DJ ^3^, Clinical investigation of subjects

Baraniuk JN ^1,^*, Organization of study and selection of samples, preparation of manuscript
